# Chronic treatment with glucocorticoids does not affect egg quality but increases cortisol deposition into egg albumen and elicits changes to the heterophil to lymphocyte ratio in a sex-dependent manner

**DOI:** 10.3389/fphys.2023.1132728

**Published:** 2023-03-17

**Authors:** E. M. Oluwagbenga, V. Tetel, S. Tonissen, D. M. Karcher, G. S. Fraley

**Affiliations:** Animal Sciences, Purdue University, West Lafayette, IN, United States

**Keywords:** chronic stress, cortisol, corticosterone, egg quality, breeder ducks

## Abstract

During chronic stress, there is an initial increase in glucocorticoid (GC) levels, but they then return to low, albeit not baseline, levels. Recent studies have renewed interest in cortisol in that it may also have important roles in the stress response. The purpose of our study was to test the hypothesis that chronic treatment with low levels of either corticosterone or cortisol would alter HLR and immune organ morphometrics. Further, we wanted to determine if chronic treatment with either GC would elicit an increase in cortisol levels in egg albumen. To test our hypotheses, we implanted silastic capsules that contained corticosterone, cortisol, or empty capsules as controls (N = 5/sex/treatment). Blood serum, smears, body weights, and egg quality data were collected. Ducks were then euthanized and body weight, weights of spleens, livers, and the number of active follicles were recorded. Albumen GC levels were assessed using mass spectrometry. Data were analyzed using a 2- or 3-way ANOVA as appropriate and post-hoc with Fishers PLSD. No treatment elicited differences in egg quality measures or body weight compared to controls. Corticosterone treatment did elicit an increase in serum corticosterone (*p* < 0.05), but not cortisol, levels compared to controls in both sexes. Both cortisol and corticosterone treatments increased (*p* < 0.05) serum levels of cortisol compared to controls. Relative spleen weights were higher (*p* < 0.05) in hens following corticosterone but not cortisol treatment. No other organs showed any differences among the treatment groups. Both GCs elicited an increase (*p* < 0.001) in HLR in hens at all time-points over the 2-week treatment period compared to controls. Cortisol, not corticosterone, only elicited an increase in HLR for drakes (*p* < 0.05) compared to controls but only at day 1 after implants. Chronic treatment with cortisol, but not corticosterone, elicited an increase (*p* < 0.01) in egg albumen cortisol levels compared to other groups. Corticosterone was not detected in any albumen samples. Our results suggest that glucocorticoids elicit differential effects and although corticosterone has been stated to be the predominant GC in avian species, cortisol may provide critical information to further understand bird welfare.

## 1 Introduction

Glucocorticoids (GC) are steroid hormones that include cortisol and corticosterone, and their secretion is controlled by the anterior pituitary hormone, adrenocorticotropic hormone (ACTH) (Salem et al., 1970). In birds, ACTH secretion is controlled by both corticotropin-releasing hormone (CRH) and arginine vasotocin (AVT) secreted from the hypothalamus (Kuenzel et al., 2012; Kang and Kuenzel, 2014; Nagarajan et al., 2014; Kang et al., 2018). Poultry experience some stressors that have been reported to elevate circulating serum GC levels (reviewed by Scanes, 2016). The hypothalamus and anterior pituitary control the release of plasma levels of corticosterone and cortisol and this is known as the hypothalamic pituitary axis (HPA). Stress activates the HPA system to cause the release of GC which in turn initiates metabolic processes directed to maintain physiological homeostasis.

For decades, researchers have investigated the effect of the body’s response to stressors that includes elevation in the level of plasma GC. These effects include stimulating gluconeogenesis and alteration of immune system functions, including leukopenia and heterophilia (Shini et al., 2008; Vicuña et al., 2015). In particular, the heterophil to lymphocyte ratio (HLR) is an indirect measure of GC function in birds. Prolonged periods of increased circulating levels of GC compromise the immunity, welfare, health, performance, and overall productivity of the bird resulting in economic losses and a decline in product quality (Post et al., 2003; Quinteiro-Filho et al., 2012; Zeng et al., 2014; Honda et al., 2015). The potential for elevated circulating levels of GC to mediate detrimental effects on production and performance parameters can be achieved through the direct introduction of the GC to the birds *via* implantation (Hayward and Wingfield, 2004), injection, oral administration in feed or water (Shini and Kaiser, 2009; Kim et al., 2015), or through the direct introduction of stimuli such as heat (Videla et al., 2020; Oluwagbenga et al., 2022), transportation (Vosmerova et al., 2010; Tetel et al., 2022b), or feed restriction (Najafi et al., 2015) to alter the HPA axis and stimulate the release of GC.

It was previously believed that corticosterone, produced by the adrenal gland, was the main GC found in birds’ plasma (deRoos, 1961). However, newer studies suggest that cortisol may also have physiological functions in poultry (Caulfield and Padula, 2020; Tetel et al., 2022b; Tetel et al., 2022a; Oluwagbenga et al., 2022) but there is a lack of research exploring the effects of both GCs, particularly cortisol, on physiological parameters and egg quality and biochemistry in ducks. It is well established that during chronic stress, there is an initial rise in glucocorticoids but after a few hours, their levels return to a low levels that still remain above baseline (reviewed by Scanes, 2016). It is not clear what physiological effects these prolonged periods of increased-baseline glucocorticoids have on the chronic stress response. Therefore, the purpose of our study was to explore the effects of prolonged exposure to increased baseline levels of corticosterone or cortisol on egg quality, HLR, and albumen GC levels. To accomplish this, we treated adult drakes and hens for 2 weeks with either GC or control using subcutaneous implants. Our results suggest that cortisol may be selectively deposited into egg albumen during periods of prolonged stress.

## 2 Materials and methods

### 2.1 Animals

Adult breeder Pekin ducks were obtained from Maple Leaf Farms Inc (Leesburg, IN) at approximately 40 weeks of age. Hens and drakes weighed 4.0–4.5 kg, respectively and we utilized five ducks of each sex per treatment, with a total of 30 ducks. The ducks were placed in floor pens in a barn at Purdue University Animal Sciences research farm with an 18:6 light cycle and *ad lib* access to water and 8 h exposure to feed per day, as per industry standards. All procedures were approved by the Purdue University Institutional Animal Care and Use Committee (PACUC #2008002065).

### 2.2 Experimental design

Pens of hens and drakes were randomly allocated into three treatments (5 ducks/treatment/sex): corticosterone, cortisol, or control. Chronic steroid treatment was delivered using subcutaneously implanted Silastic™ capsules packed with crystalline steroids. Subcutaneous implants were placed behind the neck under propofol anesthesia (2.0—8.0 mL). Each bird received two, 5 mm long 1.57 mm × 3.18 mm (inside vs. outside diameter) capsules packed with pure crystalline steroid. Decades of research using such capsules for steroid delivery have shown that release rate is related to inside and outside diameters and total, additive, length of capsules (Davidson et al., 1978; Fraley and Ulibarri, 2001; Thomas et al., 2006). Although this technique has been utilized in a great many species over the decades, it had not been used previously in ducks. Therefore, the total length of capsules had to be extrapolated based upon body weight of animals to provide the necessary dose to achieve increased basal circulating glucocorticoid levels. Controls were implanted with two empty 5 mm Silastic capsules (the vehicle for crystalline steroid delivery). Incisions were closed using tissue glue. All capsules were pre-charged in 100% ethanol before implantation to ensure immediate delivery of hormone. The use of these capsules has been established for decades as an effective delivery for constant levels of steroid hormones (Smith et al., 1977) and has been routinely used by the PI (Fraley and Ulibarri, 2001; Fraley and Ulibarri, 2002).

### 2.3 Sample collection and preparation

Blood smears were collected for HLR on days −1, 0.5, 1, 2, 4, 7, 9, 11, 13, and 14 relatives to the day of implant, with the day of implant designated as day 0 (*n* = 5/time-period/treatment). We attempted to minimize sample collection times in order to minimize handling stress while still providing a complete picture of the 2 week responses. All blood collections were completed within 30–45 s of approaching any given duck. The first post-implant blood smear was done approximately 6 h later and designated as day 0.5. Blood samples were collected from the ducks’ tibia veins on days −1, 7 and 14 relatives to subcutaneous implant into a serum separator tube, centrifuged, and the serum was stored at −20°C until assayed by ELISA for glucocorticoids (*n* = 5/time/treatment). Eggs were collected daily, combined over 3 days (*n* = 15/time-period/treatment) to reduce variability and compared among groups for egg quality assessment and albumen assay for GCs. Eggs from the 2 days preceding treatment, including the first day of treatment, were combined and labeled as group 0. Days 1, 2, and 6 were combined and labeled as group 1, days 7, 8, and 11 were combined and labeled as group 2, and days 12, 13, and 14 were combined and labeled as group 3. Albumen samples (*n* = 15/time-period/treatment) were collected into tubes during egg quality assessment and stored at −20°C until assayed for GCs using mass spectrometry.

Ducks were weighed on days −7, 1, 2, 7, and 14. After 2 weeks of GC or control exposures, all ducks were euthanized using pentobarbital (Fatal Plus, 396 mg/mL/kg) and birds were necropsied. Spleen, testes, and liver were collected, weighed, and expressed relative to the body weight (g organ weight/kg body weight). The final blood sample was obtained and treated as described above. The number of maturing follicles on the ovary were counted. Silastic capsules were removed to confirm placement and diffusion (Fraley and Ulibarri, 2001; Fraley and Ulibarri, 2002).

### 2.4 ELISA for glucocorticoids

The kits utilized for this project were from Cayman Chemicals (corticosterone: kit #16063; cortisol kit #560360) and the assays were run according to the manufacturer’s recommendations. Details of the extensive kit verification have been reported previously (Tetel et al., 2022a; Tetel et al., 2022b). Plates were incubated with samples overnight at 4°C. For the development of the plate, 250 determinations vial of Ellman’s Reagent was reconstituted with 50 mL of Ultrapure water. 200 μL of this reagent was added to each well on the plate before being placed on an orbital shaker for 90 min. At end of 90 min, plates were read at 405 nm (SynergyLx, Biotek).

### 2.5 Egg quality assessments

For the trial, eggs were collected, labelled by pen, and stored in a refrigerator at 4°C overnight. They were then weighed and their shell and vitelline membrane compression strengths were measured using a TA. XT Plus Texture Analyzer (Texture Technologies, Hamilton, MA) with a 10 kg and 500 g load cell, respectively. The procedures for these analyses have been previously described (Oluwagbenga et al., 2022). Additionally, samples of albumen were collected during the egg quality analysis and stored at −20°C for GC assays.

### 2.6 Mass spectrometry for albumen glucocorticoids

The samples were stored at −20°C before being extracted and analyzed. At the time of analysis, each albumen sample was thawed, and 500 mg was transferred to an extraction tube. The samples were extracted using a method with minor modifications, as previously reported (Caulfield and Padula, 2020). To each sample, an internal standard mixture containing 5 ng of deuterated corticosterone and 1 ng of deuterated cortisol was added and vortexed for 1 min. The samples were then derivatized with Amplifex keto reagent and analyzed by LC/MS/MS, following the kit directions and the previously reported details of extraction and analysis (Oluwagbenga et al., 2022).

### 2.7 Statistical analyses

Statistics were run using JMP Pro v.15 (SAS Institute, Cary, NC United States). For all duck-related variables, the duck was considered the statistical unit. An *a priori* Power analysis showed that our duck level sample sizes would provide 85% Power at alpha = 0.05 for the three treatment groups and two sexes. For egg quality assessment, eggs were pooled over 3 days interval (approximately N = 15/treatment/time point) and averaged. All data were analyzed by 2 -way ANOVA or repeated measures as appropriate. *Post hoc* analyses were done by Fisher’s PLSD test. A *p* < 0.05 was considered significant.

## 3 Results

### 3.1 ELISA for serum GCs

**FIGURE 1:**
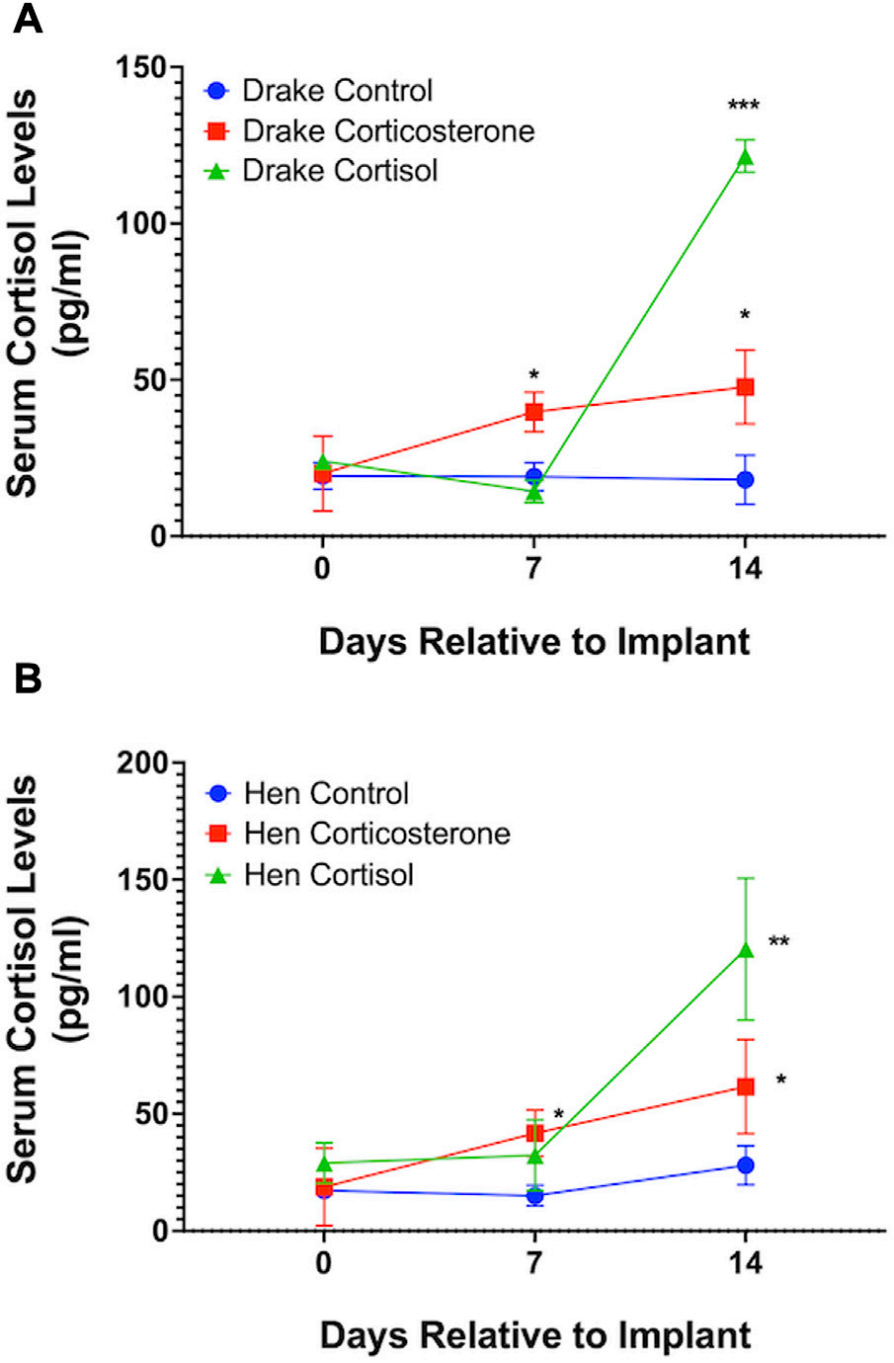
Serum cortisol levels in Drakes **(A)** and Hens **(B)**. Ducks treated with either cortisol or corticosterone showed a significant increase in serum cortisol levels compared to controls 7 and 15 days after the onset of treatment. * = *p* < 0.05, ** = *p* < 0.01, *** = *p* < 0.001.

**FIGURE 2:**
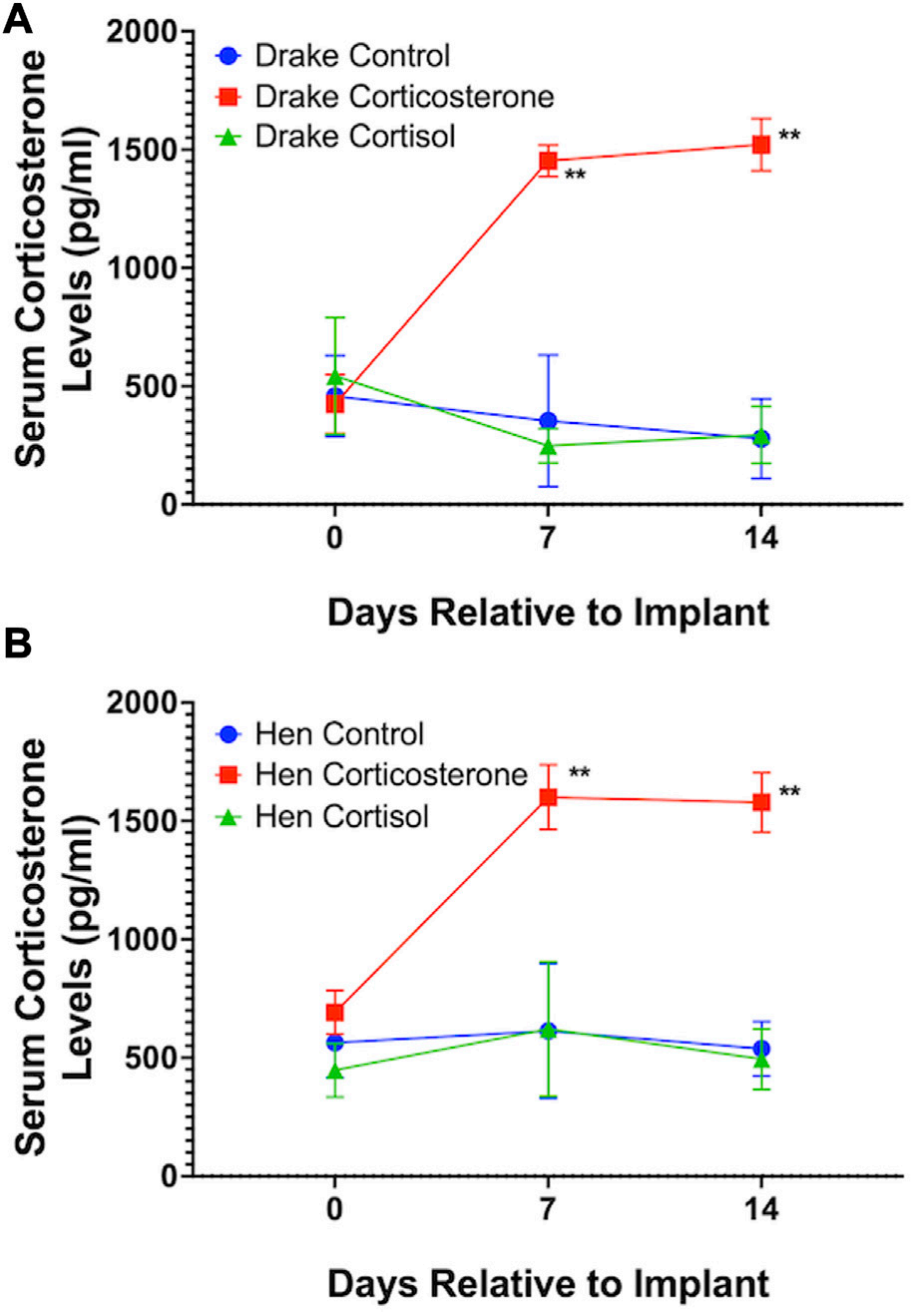
Serum corticosterone levels in Drakes **(A)** and Hens **(B)**. Only ducks treated with corticosterone showed a significant increase in serum corticosterone levels compared to controls at 7 and 15 days after onset of treatment. ** = *p* < 0.01.

Assays confirmed our goal to achieve increased basal levels of circulating glucocorticoids. Circulating levels of corticosterone, but not cortisol, were elevated in both drakes and hens in the corticosterone treatment group (*p* < 0.01) compared to cortisol treatment and controls. Cortisol treatment increased circulating levels of cortisol in both sexes (*p* < 0.01) compared to corticosterone treatment and controls. Interestingly, both corticosterone and cortisol treatments resulted in elevated cortisol levels (*p* < 0.05) compared to controls in both sexes. Figures 1, 2 illustrate these results.

### 3.2 Heterophil: Lymphocyte ratio

**FIGURE 3:**
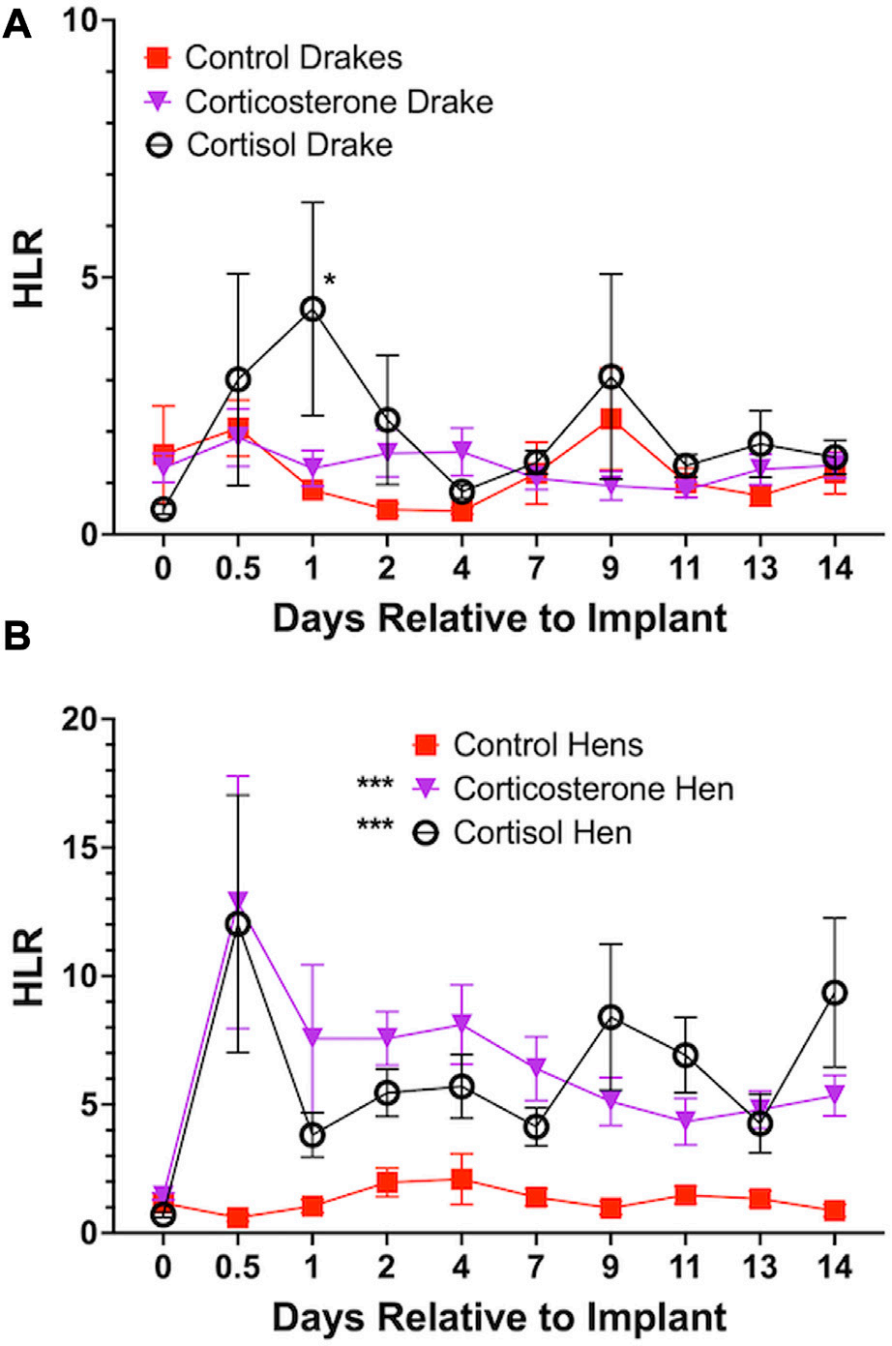
Heterophil and Lymphocyte ratio in Drakes **(A)** and Hens **(B)**. Both cortisol and corticosterone showed a significant increase in hen HLR at all time points during the experiment compared to controls. In drakes, only cortisol elicited a significant increase in HLR and only 1 day following onset of treatment compared to controls and corticosterone treatment groups. * = *p* < 0.05, *** = *p* < 0.001.

Both cortisol and corticosterone elicited an increase in HLR for hens (*p* < 0.001) beginning at day 1 and continued through the length of the experiment. Cortisol only elicited an increase in HLR for drakes (*p* < 0.05) compared to controls but only at day 1 after implants. In drakes, there were no notable increases in HLR with the corticosterone treatment. Figure 3 illustrates these results.

### 3.3 Morphometrics

There were no significant effects of corticosterone or cortisol treatments on body weights of either hens (*p* = 0.32) or drakes (*p* = 0.11). The relative spleen weight in the hens is higher in the corticosterone group (*p* < 0.01) compared to the cortisol but not the control group. Both treatments showed no significant difference in the relative liver weight for hens and the relative spleen and liver weights for drakes as shown in Table 1. Due to the age of the ducks, bursa and thymus were regressed thus we were not able to evaluate them in this study.

**TABLE 1:** Effect of a 2-week chronic stress stimulation on immune organ parameters in breeder ducks.

	Drake		Hen		
Treatment	Spleen (g/kg)	Liver (g/kg)	Spleen (g/kg)	Liver (g/kg)	Follicle (#)
Control	0.61 ± 0.068	13.3 ± 0.93	0.41 ± 0.041^ab^	21.9 ± 1.80	4.4 ± 0.68
Corticosterone	0.57 ± 0.034	14.3 ± 0.50	0.52 ± 0.038^b^	25.0 ± 0.80	4.6 ± 0.51
Cortisol	0.54 ± 0.101	13.8 ± 1.71	0.33 ± 0.025^a^	23.8 ± 1.47	5.4 ± 0.51
*p*-value	0.80	0.84	<0.01	0.34	0.45

Data shown are means ± SEM, *n* = 5/sex/treatment.

Different letter coding within parameter is significantly different (*p* < 0.05).

Values of spleen and liver are relative to the body weight (g/kg).

### 3.4 Egg quality

Results of GC treatment on egg quality are presented in Table 2. No significant effects on egg quality were observed following either GC treatment compared to controls.

**TABLE 2:** Effect of a 2-week chronic stress stimulation on egg quality parameters in breeder ducks.

Time group	Egg Weight(g)	Shell weight(g)	Haugh unit	Yolk weight(g)	Shell strength(N)	Vitelline membrane strength(N)
Control 0	91.0 ± 1.44	8.3 ± 0.09	98.8 ± 1.59	26.9 ± 0.45	51.9 ± 2.22	3.02 ± 0.359
Control 1	90.6 ± 2.20	8.1 ± 0.14	95.9 ± 1.45	25.6 ± 0.68	48.2 ± 2.45	1.94 ± 0.222
Control 2	90.1 ± 2.99	8.2 ± 0.24	96.5 ± 1.36	24.9 ± 1.07	49.5 ± 1.50	2.70 ± 0.257
Control 3	93.4 ± 2.62	8.6 ± 0.16	94.2 ± 1.97	26.1 ± 0.88	50.9 ± 2.33	2.04 ± 0.235
Corticosterone 0	87.5 ± 1.80	8.3 ± 0.30	98.6 ± 1.16	26.9 ± 0.82	52.2 ± 1.28	2.39 ± 0.268
Corticosterone 1	90.6 ± 2.00	8.3 ± 0.25	99.4 ± 1.41	27.0 ± 0.65	48.8 ± 1.59	2.58 ± 0.270
Corticosterone 2	93.3 ± 1.62	8.5 ± 0.19	100.0 ± 1.53	27.9 ± 0.73	50.5 ± 1.44	3.11 ± 0.432
Corticosterone 3	93.3 ± 3.28	8.5 ± 0.19	102.0 ± 1.96	27.6 ± 0.92	40.9 ± 6.97	3.75 ± 0.463
Cortisol 0	95.8 ± 1.28	9.0 ± 0.12	100.0 ± 1.58	29.7 ± 0.43	49.6 ± 2.78	3.21 ± 0.351
Cortisol 1	93.2 ± 1.43	8.7 ± 0.14	97.9 ± 1.13	28.5 ± 0.47	48.0 ± 3.12	2.70 ± 0.242
Cortisol 2	96.2 ± 1.02	8.9 ± 0.14	95.9 ± 1.49	28.5 ± 0.58	51.9 ± 1.68	2.92 ± 0.300
Cortisol 3	95.2 ± 1.55	9.1 ± 0.16	92.9 ± 1.65	28.2 ± 0.66	52.1 ± 3.32	2.28 ± 0.386
*p*-value	0.46	0.95	0.06	0.39	0.25	0.10

Data shown are means ± SEM, n = 15/time-period/treatment.

### 3.5 Mass spectrometry for albumen GCs

**FIGURE 4:**
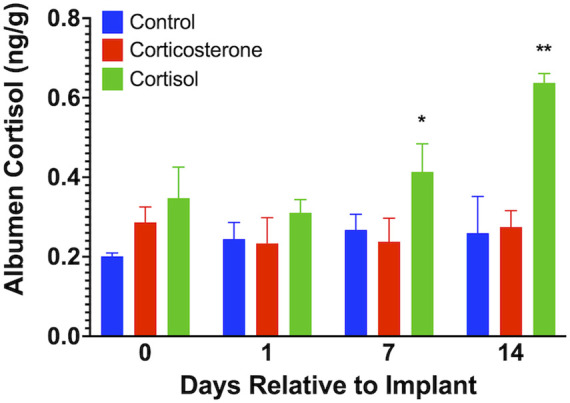
Egg albumen concentration of cortisol. Corticosterone was not detected in any samples. Albumen cortisol levels were significantly increased at 7 and 15 days after cortisol implant, but not corticosterone, treatment compared to controls. * = *p* < 0.05, ** = *p* < 0.01.

We found no measurable levels of corticosterone in eggs from any of the treatment groups similar to that previously described by our lab and others (Caulfield and Padula, 2020; Oluwagbenga et al., 2022). Albumen cortisol levels (Figure 4) were increased on day 7 (*p* < 0.05) and day 14 (*p* < 0.01) in the cortisol treatment group compared to both corticosterone and control groups.

## 4 Discussion

The purpose of our study was to explore the effects of prolonged exposure to increased circulating levels of corticosterone or cortisol on egg quality, HLR, and albumen GC levels. To accomplish this, we treated adult drakes and hens with each GC for 2 weeks using subcutaneous implants. We found no effects on any variable associated with egg quality. We reported a sex difference in the HLR whereas hens showed increased HLR following both GC treatments while drakes had a transient increase in HLR following cortisol. We found an increase in cortisol in albumen following cortisol treatment that suggests either a selective transport of cortisol into the albumen by oviduct tissues or GC-mediated *de novo* synthesis of cortisol in ovarian or oviduct tissues. Regardless, our data suggest that albumen cortisol may be an indicator of welfare or of at least chronic stress.

We have now consistently observed a sex difference in the HLR response in several studies from our lab. Tetel et al. (2022a), Tetel et al. (2022b) reported a sex-dependent increase in HLR in Pekin ducks treated with intramuscular injections of ACTH. Similarly, numerous studies have reported sex differences in the levels of GC in mammals in that female levels are higher than in males. Further, Owen et al. (2005) reported a sex difference in leukocyte composition of breeding southwestern willow flycatcher. Shini et al. (2008) reported an increase in HLR response following oral administration of corticosterone in broilers. Further, the exposure to exogenous corticosterone elevated HLR response in Japanese quail (Nazar et al., 2012) and Eurasian kestrel nestlings (Müller et al., 2011) compared to controls. It has been established that GC influences the production of lymphocytes from lymphoid tissues and cells (Weston et al., 1972; Pardue and Thaxton, 1984; Claman, 2010). In addition, several studies reported that the increase in HLR in response to various stressors can be attributed to the elevated GC levels (reviewed by (Scanes, 2016)). Therefore, sex differences in HLR ratio might be positively correlated to sex differences in the circulating levels of GC. Madison et al. (2018) demonstrated sex differences in hippocampal responses to stressors in zebra finches. These authors further stated that females showed upregulated hippocampal mineralocorticoid receptors while males downregulated both mineralocorticoid and GC receptors in response to social stressors. Sex differences could be explained by relative circulating levels of gonadal hormones. Estradiol is a circulating gonadal steroid that exerts modulating effects on HPA responsiveness and sensitivity to stress and GC negative feedback respectively (Young, 1995a; Young, 1995b). Other authors have also demonstrated that estradiol affects GC receptor effectiveness and stimulates the HPA axis (Kitay, 1963; Leśniewska et al., 1990; Xiao et al., 1994) reviewed by (Kudielka and Kirschbaum, 2005). These observations help to clarify why hens have a higher circulating level of GC and in turn HLR response to stressors and this confirms our result that showed a greater HLR in hens in response to cortisol and corticosterone administration compared to drakes.

Stress has been shown to have adverse effects on egg production and the quality of laying hens. A study from our lab showed that exposure to heat stress decreased daily egg production and decreased the shell and yolk weight of pekin ducks (Oluwagbenga et al., 2022). Both acute and chronic stress reduces egg weight and shell strength and weight, albumen deposition, yolk weight and Haugh unit (Mashaly et al., 2004; Ebeid et al., 2012; Ma et al., 2014; El-Tarabany, 2016; Barrett et al., 2019). This decrease in egg quality observed in heat-stressed ducks was attributed to the reduction in feed intake, respiratory alkalosis, and reduction in blood flow to the oviduct (Ma et al., 2014). Ebeid et al. (2012) demonstrated that heat stress leads to respiratory alkalosis due to hyperventilation and the increased blood pH reduces the amount of Ca^+^ that is essential for shell formation. The decreases in yolk and albumen quality were attributed to compromised oviduct and ovary (El-Tarabany, 2016). However, others have shown no effects of GC on egg or albumen weight (Wolfenson et al., 2007; Deng et al., 2012). Interestingly, Kim et al. (2015) studied the effect of dietary corticosterone on egg quality and reported no effects on egg weight, shell strength or weight. In our study, we found no GC effects on egg quality. Our GC treatments elicited a significant increase in circulating GC levels, but at relatively low levels indicative of GC levels following prolonged, chronic stressors. Our study did not elicit the immediate high levels of GC typical to the onset of a stressor. However our low, albeit significant, levels of GC may not have been sufficient to elicit changes in egg quality. We have previously suggested that ACTH may have extra-adrenal actions (Tetel et al., 2022b; Tetel et al., 2022a). Thus, ACTH may act directly on the oviduct or ovarian tissues to alter egg quality which was not possible in our study due to negative feedback of GCs on the diencephalon and the anterior pituitary secretion of ACTH. ACTH receptor distribution has been shown to go beyond the adrenal gland. The melanocortin two receptor (MC2R) and melanocortin five receptor (MC5R) are recognized as ACTH receptors in the adrenal gland where they regulate GC and mineralocorticoid production (Nimura et al., 2006; Mountjoy, 2010). The MC2R mRNA has been identified in the brain and blood cells of fetal mice, as well as in the brain of teleost fish (Klovins et al., 2004; Nimura et al., 2006) and in the ovary and testes of rainbow trout fish (Aluru and Vijayan, 2008). It is suggested that the MC2R found in teleost fish brains may play a role in regulating ACTH secretion through a negative feedback mechanism (Klovins et al., 2004; Nimura et al., 2006). The effect of GC on egg quality may be mediated by other receptors such as MC2R and MC5R or may be dependent on the direct action of ACTH on the oviduct. Our findings suggest that perhaps ACTH can act on oviduct tissues to impair egg quality, as evidenced by the increase in cortisol in albumen.

It was previously thought that corticosterone, produced by the adrenal cortex, is the primary plasma GC in birds (deRoos, 1961). Injecting corticosterone subcutaneously was found to increase corticosterone in the egg, providing a potential non-invasive method for measuring stress in chickens (Downing and Bryden, 2008). However, a recent study from our lab, showed that cortisol, not corticosterone, is deposited in the albumen (Oluwagbenga et al., 2022) as suggested by previous study (Caulfield and Padula, 2020). Further, we are unable to localize any glucocorticoid in the yolk (Oluwagbenga, et al., 2022). Others have demonstrated that antibody-based assays for glucocorticoids may actually cross-react with gestagens and/or pregnenalone that may elicit false-positive results (Rettenbacher et al., 2009). Steroidogenic enzymes were studied in the Bursa of Fabricius and thymus, and it was found that steroidogenic pathways within these organs lead to the synthesis of cortisol, not corticosterone (Lechner et al., 2001). Those authors further noted that cortisol has a higher affinity to the GC receptor than corticosterone in both the bursa and thymus of chickens. The increase in circulating cortisol in our study following corticosterone treatment could be explained by the corticosterone-stimulated synthesis and release of cortisol by adrenal or extra-adrenal tissues. However, confirmation of this exciting possibility requires more research. Our findings support the concept that cortisol is a key part of the stress response in ducks and that it can be selectively deposited in egg albumen, potentially serving as a non-invasive indicator of welfare.

In conclusion, our findings further confirm that there are sex differences in the HLR response of ducks to subcutaneous GC implantation where hens exhibit a greater response. We also determined that cortisol is selectively found in the egg albumen but not the yolk following implantation. Our findings suggest that GCs have diverse effects, and although corticosterone is generally considered to be the main GC in birds, cortisol may provide important insights for improving their welfare. Finally, the measurement of cortisol in egg albumen could serve as a non-invasive marker of stress, however more research must be done to affirm this possibility.

## Data Availability

The raw data supporting the conclusion of this article will be made available by the authors, without undue reservation.
